# Congenital bipartite lunate presenting as a misdiagnosed lunate fracture: a case report

**DOI:** 10.1186/1752-1947-5-102

**Published:** 2011-03-14

**Authors:** Brian WZ Loh, Jason Harvey, Eugene TH Ek

**Affiliations:** 1 Department of Orthopaedics, Dandenong Hospital (Southern Health), Dandenong Victoria 3175, Australia

## Abstract

**Introduction:**

A rare case of congenital bipartite lunate in a child is reported. Carpal variants are very uncommon as independent entities, with only three previous reports of this condition in the English literature.

**Case presentation:**

An 11-year-old Caucasian boy presented with pain in the left wrist after a fall. Radiographs in the emergency department demonstrated a lunate that was divided into palmar and dorsal parts, causing a misdiagnosis of fractured lunate. Magnetic resonance imaging was then used to differentiate between the two diagnoses.

**Conclusion:**

Very few cases of bipartite lunate have been reported in the literature, and unless awareness is raised about congenital anomalies such as this variant, confusion may arise.

## Introduction

The bipartite lunate is a rare congenital variation of the carpal bones. While anomalies such as a bipartite scaphoid, bipartite hamate and carpal synostosis are well described in the literature [1-4], to our knowledge only three cases involving the lunate have previously been reported [5-7]. Here we demonstrate the radiographic imaging from an interesting case of congenital bipartite lunate that was initially misdiagnosed as a fracture in the context of trauma.

## Case presentation

**Figure 1:**
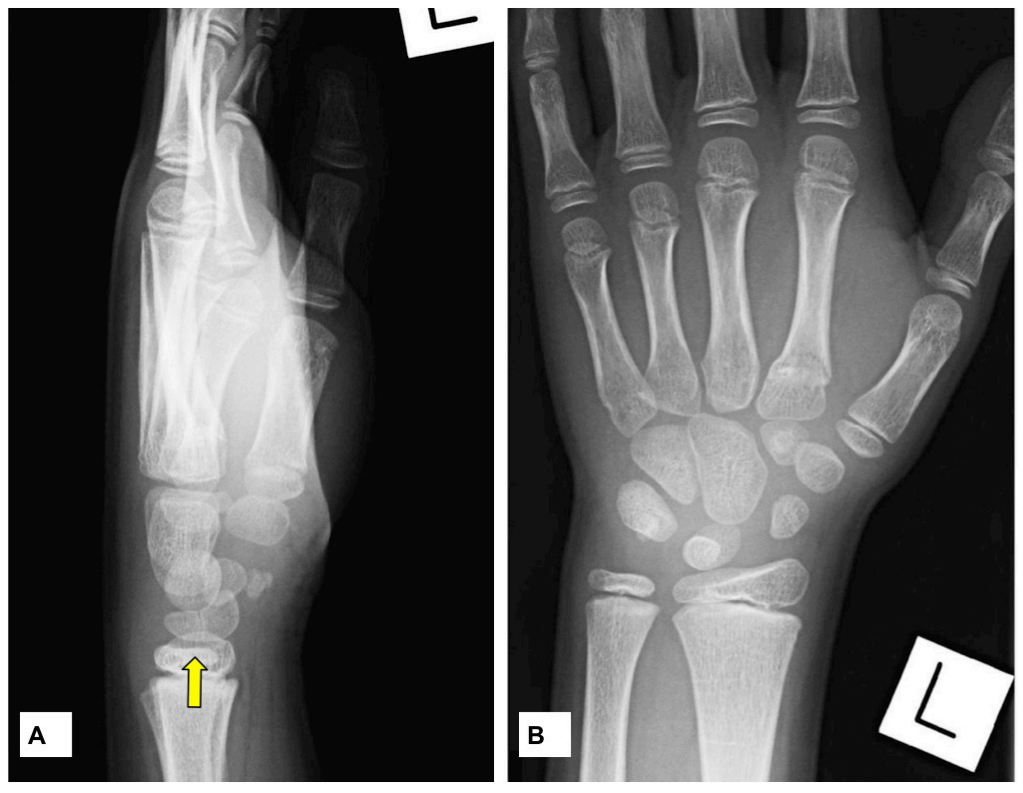
**(A) Lateral and (B) posteroanterior radiographs of the patient's left hand**. The bipartite lunate is clearly visible on the lateral radiograph (A; arrow). The margins of both lunate portions are smooth and well corticated.

An 11-year-old Caucasian boy presented to the emergency department after a fall onto the outstretched left hand. Standard posteroanterior and lateral radiographs of the wrist were performed, which demonstrated a lucent line through the lunate. On examination, the patient reported pain over the dorsum of the distal radius and in the anatomical snuffbox, but no point tenderness over the lunate. On the basis of this clinical presentation, he was provisionally diagnosed with a fractured lunate and an undisplaced scaphoid fracture (Figure 1). A scaphoid plaster cast was applied, and the patient was discharged from the emergency department. At a subsequent fracture clinic review, closer inspection of radiographs revealed that the lunate was in two distinct parts (palmar and dorsal) and that the undisplaced lunate fragments were well corticated with smooth margins.

**Figure 2:**
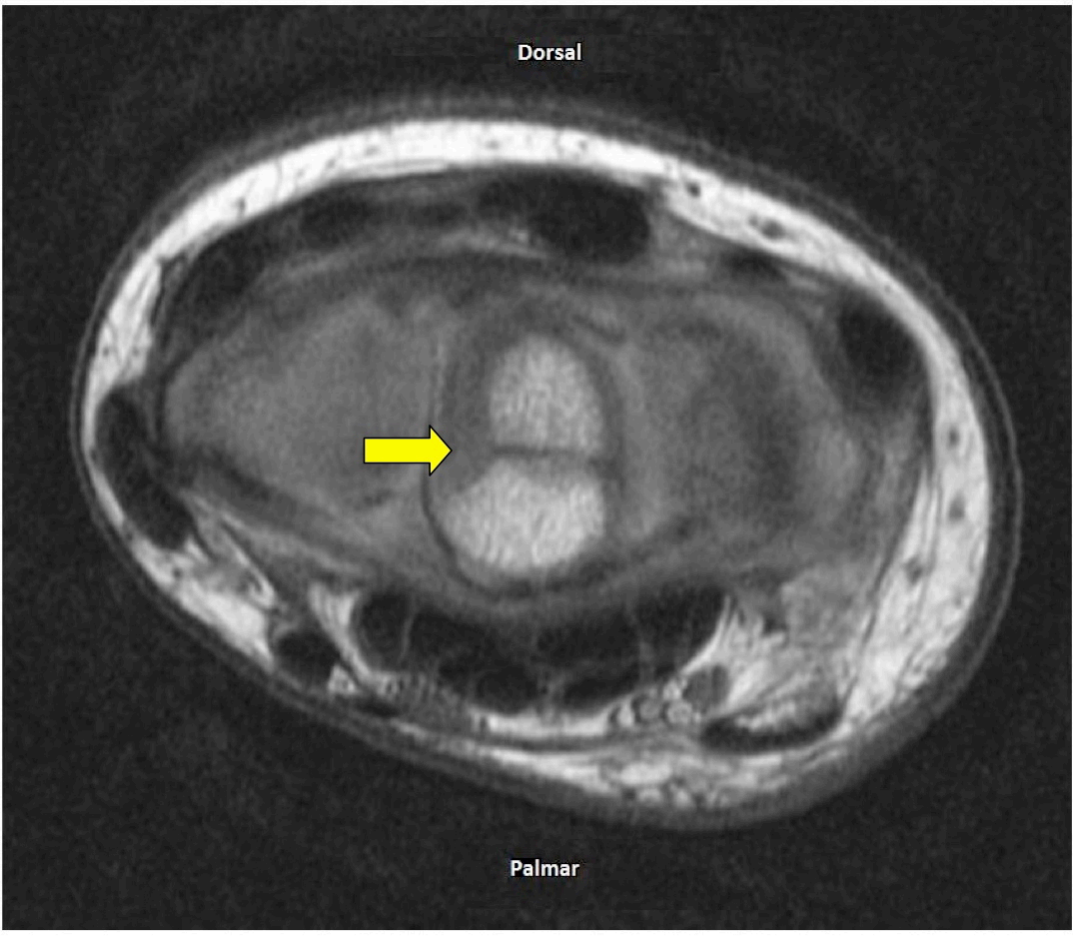
**Axial T1-weighted magnetic resonance imaging (MRI) scan of the left wrist demonstrating the linear hypointense signal (arrow) between the dorsal and palmar parts of the lunate**.

**Figure 3:**
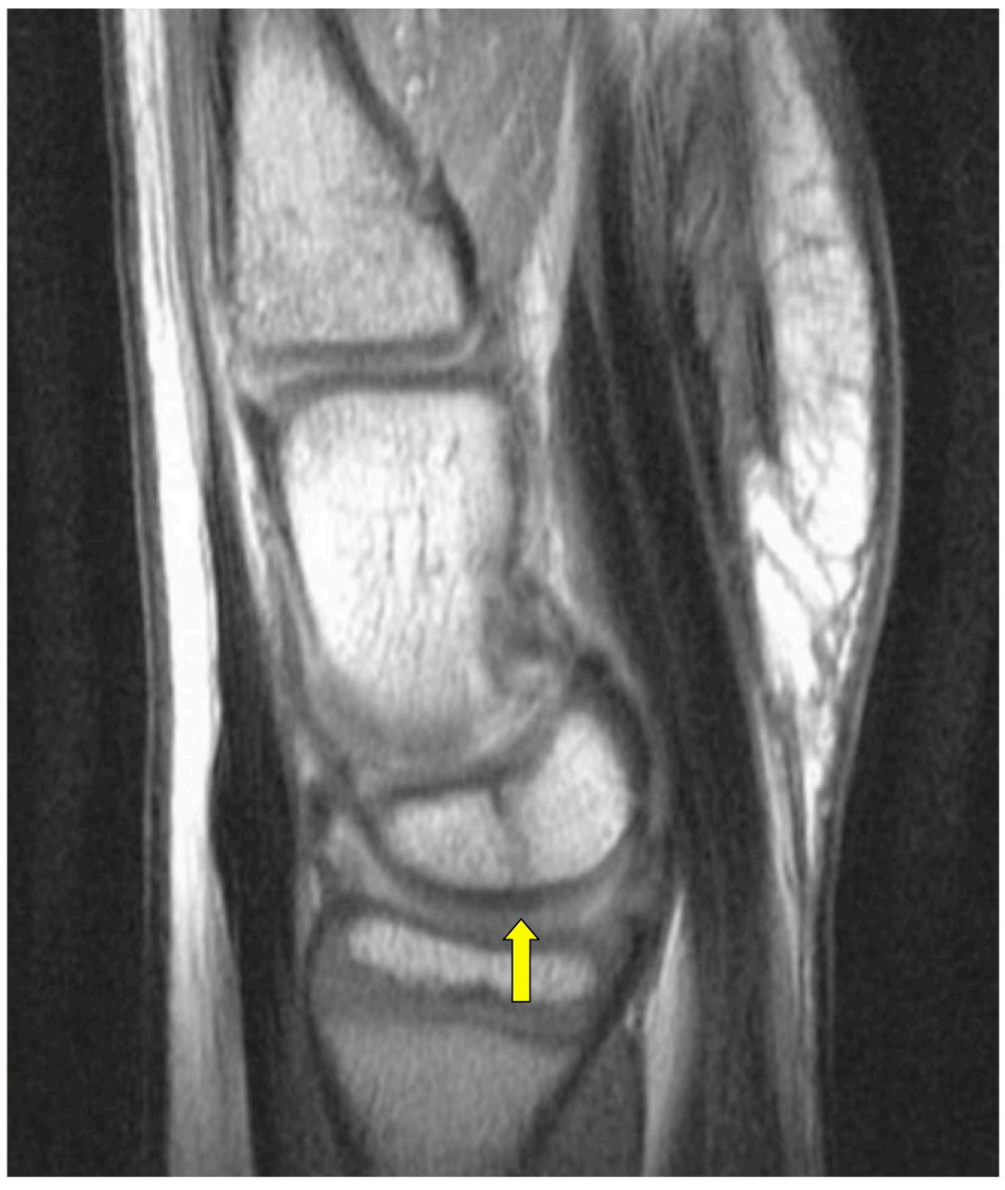
**T1-weighted MRI scan of the wrist in the sagittal plane**. The divided lunate (arrow) aligns well with the capitate and the radial articular surface.

Subsequent magnetic resonance imaging (MRI) was performed to differentiate between a traumatic lunate fracture, a bipartite congenital variant of the lunate or an atypical presentation of Kienböck's disease. Axial MRI images correlated with the plain radiographs showed a linear hypointense signal between the well-defined rounded bony margins of the palmar and dorsal parts of the lunate (Figure 2). In the sagittal plane, the divided lunate aligned well with the capitate and the articular surface of the distal radius (Figure 3). The fat-suppressed MRI images of the carpus did not show features of recent bony injury such as bone marrow edema. Furthermore, there were no features of avascular necrosis around the lunate components. An undisplaced Salter Harris type 3 fracture of the distal radius was noted, but no scaphoid fracture was observed. On a plain radiograph of the opposite wrist, no partition of the lunate was noted. The patient was treated nonoperatively with cast immobilization. At the one-year follow-up visit, the patient reported a complete recovery with no pain, normal power and a return to full range of motion.

## Discussion

The lunate begins to chondrify at Streeter's 18th and 19th horizons of embryonic development [8]. It usually has one center of ossification that appears by the age of two years, but double-ossification centers in the lunate have been noted [8]. Variation in lunate ossification is known, with ossification beginning between the ages of one and a half to seven years in boys and between one and six years of age in girls until completion between the ages of 12 to 14 for both genders [9].

As previously mentioned, congenital bipartite lunate is a rare occurrence and may lead to diagnostic difficulties in the setting of acute wrist trauma and pain. However, we did not consider the divided lunate to be a fracture, because the MRI and plain radiograph images showed that each part was well corticated with no evidence of callus formation. We concluded that our case was also unlikely to be pseudoarthrosis after a previous lunate fracture because there was neither a history of trauma to the wrist nor evidence of degenerative change. Last, the patient did not display any radiographic signs suggestive of Kienböck's disease, such as lunate fragmentation due to devascularized, necrotic bone and decreased T1-weighted signal intensity on MRI images [8].

## Conclusion

Injuries to the wrist and carpal bones are extremely common, and unless there is awareness about congenital anomalies such as the bipartite lunate, scaphoid and hamate, confusion and misdiagnoses may arise.

## Competing interests

The authors declare that they have no competing interests.

## Consent

Written informed consent was obtained from the parents of the patient for publication of this case report and accompanying images. A copy of the written consent is available for review by the Editor-In-Chief of this journal.

## Authors' contributions

EE identified the patient and performed the initial investigation and examination. BL undertook further investigation and wrote the manuscript. JH was the supervising consultant and was a major contributor in writing the manuscript. All authors read and approved the final manuscript.
